# Sensations in Drowning

**Published:** 1854-12

**Authors:** F. Beaufort


### SENSATIONS IN DROWNING.

To the many thousands scattered throughout this wide land, whose hearts were wrung by the loss of the Arctic, where, in five hours, three hundred souls, numbering among them some of the brightest and best spirits of our times, perished amid the remorseless waters, the following letter of Admiral Beaufort to Dr. Wollaston, and originally published in the life of Sir John Barrow, may be read with interest:—

"The following circumstances which attended my being drowned have been drawn up at your desire; they had not struck me as being so curious as you consider them, because from two or three persons, who, like myself, have been recovered from a similar state, I have heard a detail of their feelings, which resemble mine as nearly as was consistent with our different constitutions and dispositions.

"Many years ago, when I was a youngster on board one of his Majesty's ships in Portsmouth Harbor, after sculling about in a very small boat, I was endeavoring to fasten her alongside the ship to one of the scuttle-rings; in foolish eagerness I stepped upon the gunwale; the boat, of course, upset, and I fell into the water, and not knowing how to swim, all my efforts to lay hold either of the boat or the floating sculls were fruitless. The transaction had not been observed by the sentinel on the gangway, and therefore it was not until the tide had drifted me some distance astern of the ship that a man in the foretop saw me splashing in the water and gave the alarm. The first lieutenant instantly and gallantly jumped overboard, the carpenter followed his example, and the gunner hastened into a boat and pulled after them. With the violent but vain attempts to make myself heard I had swallowed much water; I was soon exhausted by my struggle, and before my relief reached me, I had sank below the surface; all hopes had fled —all exertion ceased—and I *felt* that I was drowning.

"So far, these facts were either partially remembered after my recovery or supplied by those who had latterly witnessed the scene; for during an interval of such agitation a drowning person is too much occupied in catching at every passing straw, or too much absorbed by alternate hope and despair, to mark the succession of events very accurately. Not so, however, with the facts which immediately ensued; my mind had been undergoing the sudden revolution which appeared to you so remarkable, and all the circumstances of which are now as vividly fresh in my memory as if they had occurred but yesterday. From the moment that all exertion had ceased—which I imagine was the immediate consequence of complete suffocation—a calm feeling of the most perfect tranquillity superseded the previous tumultuous resignation—for drowning no longer appeared to be an evil—I no longer thought of being rescued, nor was I in any bodily pain. On the contrary, my sensations were now of rather a pleasurable cast, partaking of that dull but contented sort of feeling which precedes the sleep produced by fatigue. Though the senses were thus deadened, not so the mind; its activity seemed to be invigorated in a ratio which defies all description, for thought rose after thought with a rapidity of succession that is not only indescribable, but probably inconceivable by any one who has not himself been in a similar situation. The course of those thoughts I can even now in a great measure retrace; the event which had just taken place—the awkwardness that had produced it—the bustle it must have occasioned (for I had observed two persons jump for the chains)—the effect it would have on a most affectionate father—the manner in which he would disclose it to the rest of the family—and a thousand other circumstances minutely associated with home, were the first series of reflections that occurred. Then they took a wider range—our last cruise—a former voyage, and shipwreck—my school—the progress I made there, and the time I had misspent—and even all my boyish pursuits and adventures. Thus travelling backwards, every past incident of my life seemed to glance across my recollection in retrograde succession; not however, in mere outline, as here stated, but the picture filled up with every minute and collateral feature; in short, the whole period of my existence seemed to be placed before me in a kind of panoramic review, and each act of it seemed to be accompanied by a consciousness of right or wrong, or by some reflection on its cause or its consequences; indeed many trifling events which had been long forgotten then crowded into my imagination, and with the character of recent familiarity. May not all these be some indication of the almost infinite power of memory with which we may awaken in another world, and thus be compelled to contemplate our past lives? But however that may be, one circumstance was highly remarkable; the innumerable ideas which flashed into my mind were all retrospective; yet I had been religiously brought up; my hopes and fears of the next world had lost nothing of their early strength, and at any other period intense interest and awful anxiety would have been excited by the mere probability that I was floating on the threshold of eternity; yet at that inexplicable moment, when I had full conviction that I had crossed that threshold, not a single thought wandered into the future—I was wrapt entirely in the past. The length of time that was occupied by this deluge of ideas, or rather the shortness of time into which they were condensed, I cannot now state with precision, yet certainly two minutes could not have elapsed from the moment of suffocation to that of my being hauled up.

"The strength of the flood-tide made it expedient to pull the boat at once to another ship, where I underwent the usual vulgar process of emptying the water by letting my head hang downwards, then bleeding, chafing, and even administering gin; but my submersion had been really so brief, that according to the account of the lookers on, I was very quickly restored to animation.

"My feelings while life was returning were the very reverse in every point of those which have been described above. One single but confused idea—a miserable belief that I was drowning—dwelt upon my mind, instead of the multitude of clear and definite ideas which had recently rushed through it, a helpless anxiety—a kind of continuous nightmare—seemed to press heavily on every sense, and to prevent the formation of any distinct thought, and it was with difficulty that I became convinced that I was really alive. Again, instead of being absolutely free from bodily pain, as in my drowning state, I was now tortured with pain all over me; and though I have since been wounded in several places, and have often submitted to severe surgical discipline, yet my sufferings were at that time far greater; at least, in general distress. On one occasion I was shot in the lungs, and, after lying on the deck at night for some hours bleeding from other wounds, I at length fainted. Now, as I felt sure that the wound in the lungs was mortal, it will appear obvious that the overwhelming sensation which accompanies fainting must have produced a perfect conviction that I was then in the act of dying. Yet nothing in the least resembling the operations of my mind when drowning then took place; and when I began to recover, I returned to a clear conception of my real state.

"If these *involuntary experiments* on the operation of death afford any satisfaction or interest to you they will not have been suffered quite in vain by

"Yours very truly, F. Beaufort."

